# Trends in corneal transplantation at the University Eye Hospital in Tübingen, Germany over the last 12 years: 2004 – 2015

**DOI:** 10.1371/journal.pone.0198793

**Published:** 2018-06-25

**Authors:** Tobias Röck, Karl U. Bartz-Schmidt, Daniel Röck

**Affiliations:** Centre for Ophthalmology, Eberhard-Karls-University Tübingen, Tübingen, Germany; Central Clinical School, University of Sydney Sydney Law School, AUSTRALIA

## Abstract

**Purpose:**

This study aimed to investigate the trends in the surgical methods and leading indications for corneal transplantations carried out over the last 12 years.

**Methods:**

The data from the corneal graft waiting list and from all keratoplasties carried out between 2004 and 2015 at the University Eye Hospital in Tübingen were retrospectively analyzed.

**Results:**

A total of 1,185 keratoplasties were performed between 2004 and 2015 at this hospital. The most common surgical indications for corneal transplantation were Fuchs’ endothelial corneal dystrophy (35.2%) and keratoconus (18.9%) with keratoconus being the leading cause during early years (from 2004 to 2009) and Fuch’s dystrophy being the leading cause from 2010 to 2015. Overall, the total count of performed keratoplasties increased, from 385 corneal transplantations during the first 6-year period to 800 corneal transplantations during the second 6-year period (P = 0.008, using Mann-Whitney test). The Descemet’s membrane endothelial keratoplasty has become the favored surgical method for endothelial disorders with the number of Descemet’s membrane endothelial keratoplasties increasing significantly from 2008 to 2015. This increasing trend was statistically significant (P < 0.001 using multivariate adaptive regression splines (MARS). A decreasing trend was also noted for the rate of penetrating keratoplasty since 2008 (P < 0.001 using MARS).

**Conclusions:**

This research showed major changes in the preferred corneal transplantation techniques and leading indications for keratoplasty over the last 12 years. More importantly, it seems that the rapid development and implementation of endothelial keratoplasty, especially the Descemet’s membrane endothelial keratoplasty, has had a profound effect on and begun a new era in corneal transplantation.

## Introduction

Keratoplasties have been performed successfully in humans for over a hundred years [1], and are the most frequent type of transplantation performed in human beings. In 2012, 184,576 corneal transplantation procedures were performed in 116 countries [2].

In general, the development of corneal transplantation techniques has been very fast, while undergoing continuous improvement. Moreover, the development of these surgical techniques has undergone significant changes over the last 12 years, allowing for the increasingly successful replacement of selective corneal layers [3]. Unfortunately, a serious lack of corneal tissue exists in most countries [4,5]. This is largely due to the growing need for corneal grafts as a result of demographic changes, as well as the growing number of surgical procedures required, especially endothelial procedures [3,6].

In Germany, the last few years have been challenging with regard to recruiting corneal donors after several transplantation scandals [7,8]. The public has suffered a massive lack of confidence in the transplantation system, and this loss of trust has influenced the donation rate significantly [9,10]. Moreover, our study group showed that the acquisition of donor corneas is based on sufficient eye bank team staff levels [11], increasing public education about corneal donation [12], a working system of connections between intensive care units and the responsible eye bank representatives [13], the approachability of usable donors [14,15], and previous informed consent through a donor card, verbal or written consent, or through the approval of the next of kin [16].

Eleven years ago, in 2006, Darlington et al. performed a review of corneal transplantations, analyzing data from 1980 to 2004 [17]. They reported that more than 95% of the corneal tissues were used for penetrating keratoplasties (PKs), with the major indications being pseudophakic bullous keratopathy, keratoconus, Fuchs’ endothelial dystrophy (or Fuchs' dystrophy), and repeat grafts. Since then, several new lamellar keratoplasty techniques have been described: Descemet’s stripping endothelial keratoplasty (DSEK) [18], Descemet’s stripping automated endothelial keratoplasty (DSAEK) [19], and Descemet’s membrane endothelial keratoplasty (DMEK) [20]. These procedures, especially the DMEK, provide various benefits over the PK, such as minimal invasiveness, smallest chance of rejection, and minimal refractive shift with very quick visual improvement [21]. When confronted with the new possibilities of lamellar keratoplasty, and inspired by the investigation of Darlington et al. [17], we decided to investigate the changing trends in the corneal transplantation methods, and the leading indications for corneal transplantation.

## Material and methods

This study was approved by the institutional review board of the University of Tübingen, and adhered to the tenets of the Declaration of Helsinki. The study data was accessed anonymously from the annual reports of the eye bank at the University Eye Hospital in Tübingen between 2004 and 2015. Consent for use of medical records was not needed. The indications for transplantation were categorized according to keratoconus, Fuchs' dystrophy, bullous keratopathy, trauma, rejection, and others. The indications for corneal transplantation numbers were obtained from the waiting list, and collected on December 31^st^ of the respective year.

The number of keratoplasty procedures was calculated on an annual basis following the calendar year (January 1^st^ to December 31^st^). The actual keratoplasty surgical numbers each year were evaluated and compared on the basis of the surgical method [PK, DSAEK, DMEK, deep anterior lamellar keratoplasty (DALK), and Boston keratoprosthesis].

## Statistical analysis

The statistical analysis of the data was conducted using the Statistical Package for the Social Sciences version 18.0 (SPSS Inc., Chicago, IL, USA) and R studio Version 1.1.419 (250 Northern Ave, Boston, MA 02210). The quantitative variables were summarized as counts and percentages. Mann-Whitney test was used to compare the total count of keratoplasties and the percentage of operations performed by various methods (PK and DMEK) during the first half (six years) of the study to those performed during the second half. It was also used to compare the indications (percentage) across the two time periods. Mann-Whitney test was used since it does not require the data to be normally distributed and is not affected by violations to normality. Multivariate adaptive regression splines (MARS) was used to assess whether a yearly trend existed in keratoplasty rates (methods and indications) as well as the significance of such trend. The advantage of using MARS is that it is a non-parametric regression technique that automatically models non linearity in analysis which makes it suitable for use since keratoplasty rates were constant during the first few years which affects the data distribution. P<0.05 was considered to be statistically significant throughout the analysis.

## Results

Between 2004 and 2015, 1,185 keratoplasties were carried out at this institution. These included 726 (61.3%) PKs and 459 (38.7%) lamellar keratoplasties (LKs). Overall, the total count of performed keratoplasties increased, from 385 corneal transplantations during the first 6-year period to 800 corneal transplantations during the second 6-year period (P = 0.008, using Mann-Whitney test). Moreover, there was an increasing trend in the total number of keratoplasties performed (P < 0.001 using MARS).

### Surgical technique

**Fig 1:**
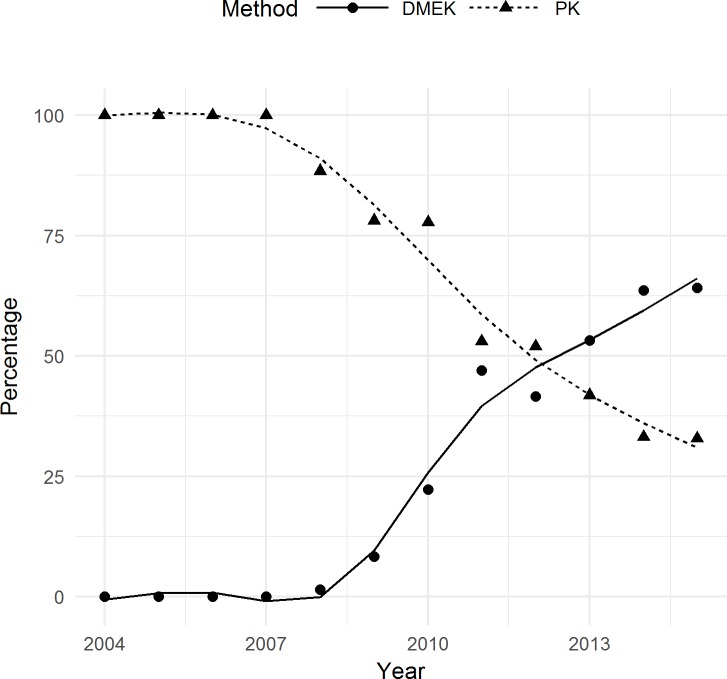
DMEKs and PKs performed from 2004 to 2015. Lines represent the trend produced by MARS. The DMEK has become the favored surgical method for endothelial disorders since 2013 (53.2% vs. 41.8% in 2013 for DMEK and PK, respectively). No DMEKs were performed from 2004 through 2007. Starting from 2008, a statistically significant increasing trend was noted for the percentage of DMEK (P < 0.001 using MARS). On the contrary, a decreasing trend was noted for the rate of PK which indicates that the percentage of PKs significantly decreased from 2008 through 2015 (P < 0.001, using MARS).

Between 2004 and 2015, the annual PK numbers remained constant. The keratoplasties carried out between 2004 and 2007 were purely in the form of PKs, with the first endothelial keratoplasty (EK) being carried out in 2008 using the DSAEK method. Twenty DSAEK procedures were carried out from 2008 to 2009. However, the DMEK quickly replaced the DSAEK at this center, and has continued as the favored EK technique until now. One DMEK procedure was carried out in 2008 (representing 1.4% of all 2008 keratoplasties), while 84 DMEKs were carried out in 2015 (representing 64.1% of all 2015 keratoplasties) (Table 1). The DMEK has become the favored surgical method for endothelial disorders since 2013 (53.2% vs. 41.8% in 2013 for DMEK and PK, respectively). No DMEKs were performed from 2004 through 2007. Starting from 2008, a statistically significant increasing trend was noted for the percentage of DMEK (P < 0.001 using MARS). On the contrary, a decreasing trend was noted for the rate of PK which indicates that the percentage of PKs significantly decreased from 2008 through 2015 (P < 0.001, using MARS) (Fig 1).

**Table 1:** Annual numbers and rates of corneal transplantations.

Year	Corneal Transplantations [n]	PK[n,(%)]	DSAEK [n,(%)]	DMEK[n,(%)]	DALK[n,(%)]	Boston Keratoprosthesis [n,(%)]
2004	50	50 (100%)	0	0	0	0
2005	53	53 (100%)	0	0	0	0
2006	48	48 (100%)	0	0	0	0
2007	69	69 (100%)	0	0	0	0
2008	69	61 (88.4%)	7 (10.1%)	1 (1.4%)	0	0
2009	96	75 (78.1%)	13 (13.5%)	8 (8.3%)	0	0
2010	90	70 (77.8%)	0	20 (22.2%)	0	0
2011	100	53 (53.0%)	0	47 (47.0%)	0	0
2012	154	80 (51.9%)	0	64 (41.6%)	10 (6,4%)	0
2013	141	59 (41.8%)	0	75 (53.2%)	7 (5.0%)	0
2014	184	61 (33.2%)	0	117 (63.6%)	6 (3.3%)	0
2015	131	43 (32.8%)	0	84 (64.1%)	0	4 (3.1%)
	1185	722 (60.9%)	20 (1.7%)	416 (35.1%)	23 (1.9%)	4 (0.3%)

This table shows the annual numbers and rates of corneal transplantations by technique (PK, DSAEK, DMEK, DALK, and Boston keratoprosthesis) from 2004 to 2015.

Analysis also showed that there was a significant difference in the rate of PKs between the two study periods (92.5% during the first six years vs. 45.8% during the second six years, P = 0.004 using Mann-Whitney test)

The Boston keratoprosthesis, DALK, and DSAEK were all carried out in smaller numbers during the 12-year period, representing 0.3%, 1.9%, and 1.7% of the total keratoplasties, respectively.

**Fig 2:**
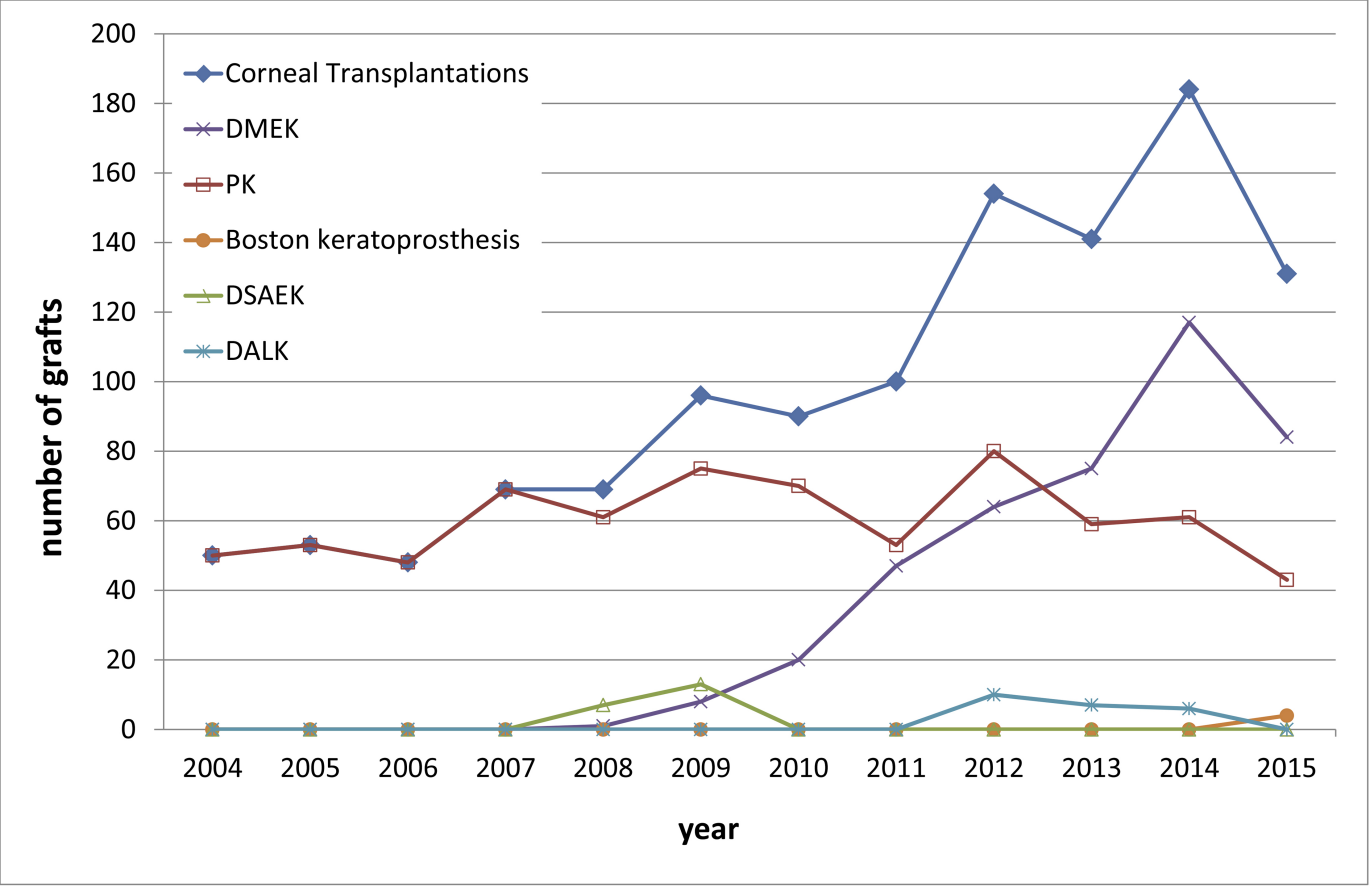
Number of grafts performed by technique. This figure shows the distribution of the corneal transplantations by technique (PK, DSAEK, DMEK, DALK, and Boston keratoprosthesis) from 2004 to 2015 in 1,185 keratoplasties. Between 2004 and 2007, the corneal transplantations consisted of PKs exclusively. The first EK was performed in 2008 using the DSAEK technique. The DMEK quickly replaced the DSAEK at our institution, and has remained the preferred surgical technique for endothelial diseases, surpassing the PK in 2013. The number of DMEKs increasing significantly as the total number of corneal transplants increased from 2008 to 2015. This increasing trend was statistically significant (P < 0.001 using MARS).

Fig 2 shows the distribution of the corneal transplantations by technique (PK, DSAEK, DMEK, DALK, and Boston keratoprosthesis) of the 1,185 keratoplasties performed from 2004 to 2015.

### Indications for corneal transplantation

**Fig 3:**
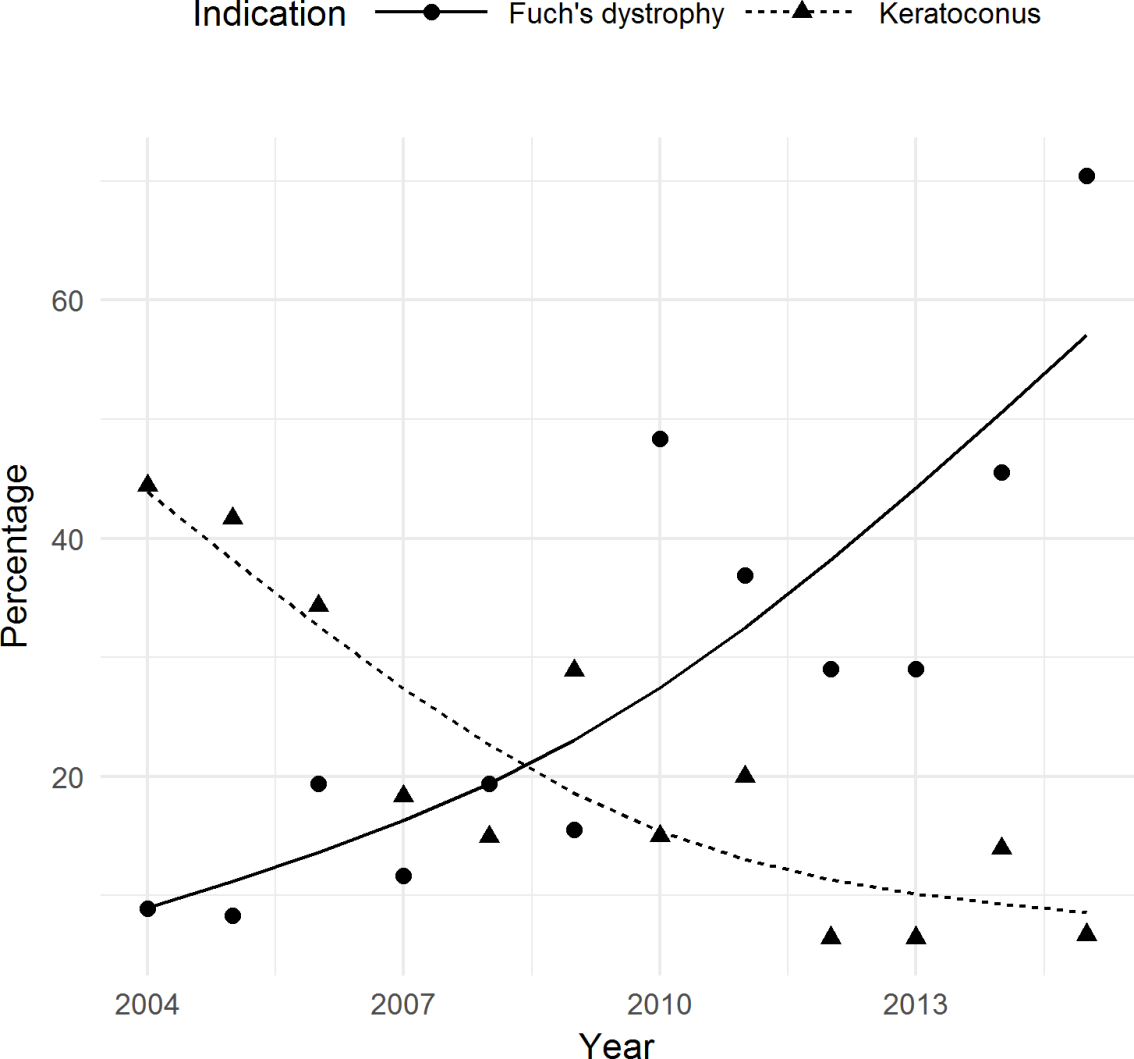
Fuch’s dystrophy and keratoconus as leading surgical indications from 2004 to 2015. Lines represent the trend produced by MARS. The percentage (rate) of Fuchs' dystrophy as a surgical indication for corneal transplantation has increased significantly from 2004 to 2015 (P < 0.001 using MARS). A deceasing trend was noted in the percentage of keratoplasties due to keratoconus (P < 0.001 using MARS) (Fig 3).

The indications for corneal transplantation numbers were obtained from the waiting list, which increased from 45 patients in 2004 to 149 patients in 2015. During the study period, the leading surgical indications for keratoplasty were Fuchs' dystrophy (35.2%) and keratoconus (18.9%) with keratoconus being the leading cause during early years (from 2004 to 2009) and Fuch’s dystrophy being the leading cause from 2010 to 2015 (Table 2). The additional indications for corneal transplantation were bullous keratopathy (10.9%), trauma (5.0%), rejection (11.4%), and others, such as bacterial, fungal, viral, or Acanthamoeba keratitis, penetrating corneal ulcers due to trophic disease, or chemical and thermal burns of the cornea (18.5%). In 2004, keratoconus was the most common indication (44.4%) for corneal transplantation, but in 2015, it was only the third most common (6.7%). Analysis showed opposite trends in keratoplasty rates due to keratoconus and Fuch’s dystrophy. The percentage (rate) of Fuchs' dystrophy as a surgical indication for corneal transplantation has increased significantly from 2004 to 2015 (P < 0.001 using MARS). A deceasing trend was noted in the percentage of keratoplasties due to keratoconus (P < 0.001 using MARS) (Fig 3). Fuchs' dystrophy was only the fifth most common indication (8.9%) for corneal transplantation in 2004, but it was the most common (70.5%) indication in 2015.

**Table 2:** Waiting list with indications for corneal transplantation.

Year	Indication for Keratoplasty [n]	Keratoconus [n,(%)]	Fuchs' dystrophy [n,(%)]	Bullous Keratopathy [n,(%)]	Trauma[n,(%)]	Rejection[n,(%)]	Others[n,(%)]
2004	45	20 (44.4%)	4 (8.9%)	7 (15.6%)	5 (11.1%)	4 (8.9%)	5 (11.1%)
2005	36	15 (41.7%)	3 (8.3%)	2 (5.6%)	3 (8.3%)	4 (11.1%)	9 (25.0%)
2006	67	23 (34.3%)	13 (19.4%)	2 (3.0%)	6 (9.0%)	11 (16.4%)	12 (17.9%)
2007	60	11 (18.3%)	7 (11.7%)	9 (15.0%)	7 (11.7%)	16 (26.7%)	10 (16.7%)
2008	67	10 (14.9%)	13 (19.4%)	14 (20.9%)	3 (4.5%)	11 (16.4%)	16 (23.8%)
2009	45	13 (28.9%)	7 (15.6%)	3 (6.7%)	4 (8.9%)	8 (17.8%)	10 (22.2%)
2010	60	9 (15.0%)	29 (48.3%)	6 (10.0%)	2 (3.3%)	5 (8.3%)	9 (15.0%)
2011	65	13 (20.0%)	24 (36.9%)	5 (7.7%)	4 (6.2%)	5 (7.7%)	14 (21.5%)
2012	31	2 (6.5%)	9 (29.0%)	3 (9.7%)	0	6 (19.4%)	11 (35.5%)
2013	31	2 (6.5%)	9 (29.0%)	3 (9.7%)	0	6 (19.4%)	11 (35.5%)
2014	79	11 (13.9%)	36 (45.6%)	6 (7.6%)	1 (1.3%)	6 (7.6%)	19 (24.1%)
2015	149	10 (6.7%)	105 (70.5%)	20(13.4%)	2 (1.3%)	2 (1.3%)	10 (6.7%)
	735	139 (18.9%)	259 (35.2%)	80 (10.9%)	37 (5.0%)	84 (11.4%)	136 (18.5%)

This table shows the annual numbers and percentages of patients on the corneal graft waiting list on December 31^st^ of each respective year with indications for corneal transplantation (Fuchs' dystrophy, keratoconus, bullous keratopathy, trauma, rejection, and others) from 2004 to 2015.

**Fig 4:**
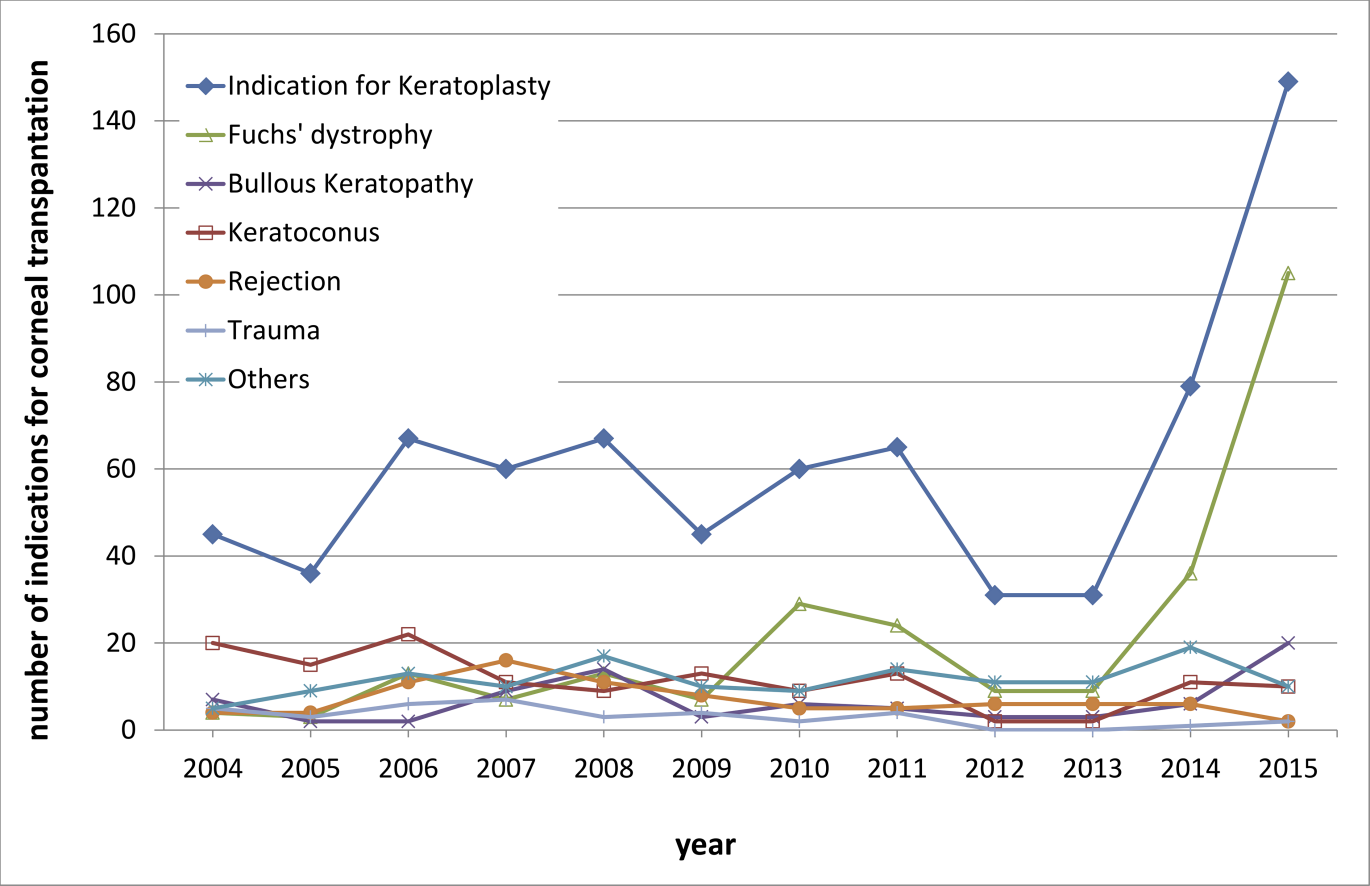
Waiting list distribution with indications for corneal transplantation. This figure shows the distribution of the corneal graft waiting list with indications for corneal transplantation (Fuchs' dystrophy, keratoconus, bullous keratopathy, trauma, rejection, and others) from 2004 to 2015 in 735 cases. The most common surgical indications for corneal transplantation were Fuchs’ endothelial corneal dystrophy (35.2%) and keratoconus (18.9%) with keratoconus being the leading cause during early years (from 2004 to 2009) and Fuch’s dystrophy being the leading cause from 2010 to 2015.

Fig 4 shows the distribution of the corneal graft waiting list with indications for corneal transplantation (Fuchs' dystrophy, keratoconus, bullous keratopathy, trauma, rejection, and others) from 2004 to 2015 in 735 cases.

## Discussion

Our study examined the 12-year trend changes in the corneal transplantation methods and leading indications for corneal transplantation at the University Eye Hospital in Tübingen, Germany. The total count of performed keratoplasties increased, from 385 corneal transplantations during the first 6-year period to 800 corneal transplantations during the second 6-year period. The main reasons for this growth rate are the demographic changes (i.e., more and more elderly individuals requiring corneal transplantations), changes in the surgical procedures (especially endothelial), and the increased corneal donation rate at this institution between 2002 and 2015 [11].

The keratoplasties carried out between 2004 and 2007 were purely in the form of PKs. The first EK was performed in our center in 2008 using the DSAEK method, with 20 DSAEK procedures being carried out from 2008 to 2009. The DMEK quickly replaced the DSAEK at our facility, and has continued as the favored EK technique to manage endothelial cell disorders until now. The well-established DMEK was first reported in 2006 by Melles [3,20], and provides several advantages over the PK, including its minimal invasiveness, lower intraoperative risks, and minimal refractive shift with very fast visual recovery [21–23]. One DMEK procedure was performed in 2008 (representing 1.4% of all 2008 transplantations), while 84 DMEK procedures were performed in 2015 (representing 64.1% of all 2015 transplantations). The number of DMEKs increasing significantly (P < 0.001).

The improvements in the microsurgical techniques, as in the EK procedure, and enormous success of the DMEK have allowed many patients with endothelial disorders to undergo keratoplasties earlier than before, if the corneal tissue is available. Corneal cases that were previously considered to be too early for PKs are now qualified for DMEKs. This is in contrast to 2006, when Afshari et al. reported that almost no eyes with Fuchs' dystrophy and visual acuities better than 0.5 received keratoplasties (especially PKs) [24]. In our eye hospital, we favor the use of patient complaints as the leading indication for the surgical treatment of Fuchs' dystrophy. When these complaints indicate that the daily activities are significantly affected, we choose DMEK treatment, even if the visual acuity is 0.6 or better. However, the surgical center needs a specialist surgeon who feels comfortable with the DMEK procedure, which requires a sophisticated technique. There is always the possibility that a surgeon is afraid to change procedures (from DSAEK to DMEK for example) when graft reserves are lacking due to the higher hazard of graft preparation failure, and consequently, graft waste when acquiring this novel method. Fortunately, our center has its own in-house eye bank and reserve corneal tissue in case of graft preparation failure.

Maier et al. and our study group reported in 2015 that an important factor for the outcome of a DMEK surgery in patients with endothelial disorders is not waiting too long to conduct the surgery [25,26]. Our results showed a relationship between the DMEK outcome and disease severity [25]. With the advances in the EK, these patients now receive and also require corneal transplantation surgeries earlier. Overall, a better visual acuity corresponds to a higher quality of life. For these reasons, the pressure on eye banks to procure more suitable corneal grafts has been growing.

The demand for eye bank corneas for EK has increased dramatically in the last years. Corneal graft scarcity is a common dilemma almost all over the world. There is a lack of corneal tissue due to the shortage of donor corneas with demographic change with aging of the population and the increasing trend towards lamellar EK procedures. Improvements in microsurgical techniques like the EK procedure and the enormous success of the DMEK has allowed for many patients with endothelial disorders as mentioned above to enable keratoplasties earlier than before. Corneas that had been considered to be too early for PK have been nowadays qualified for DMEK. Additionally, DMEK provides even faster visual rehabilitation and reduced risk of immunologic rejection, so its use is growing. For these reasons, our waiting list is growing in the last years. We wanted to express the actual situation and trend with these numbers from the corneal graft waiting list.

The most common surgical indications for corneal transplantation were Fuchs' dystrophy and keratoconus, which were almost equal in numbers to the main indications for corneal transplantation reported in former studies [27–30]. Although the DALK was described in 2002 [31], it was carried out only in small numbers during the 12-year study period, representing 2% of the total corneal grafts. The moderate introduction of DALK is probably due the higher technical challenge, lower number of patients with indications for DALK, and consequently, the slower and more difficult learning curve for the surgeon. There was a low number of indications; however, the DALK may be technically very difficult, since the risk of intraoperative anterior chamber entry increases during the stromal dissection, thus requiring an on-table conversion to a PK. Additionally, a PK is considered to be more suitable in cases in which endothelial dysfunction is present, or when deep corneal scarring severely affects the visual axis up to the DM level. Moreover, it is not unusual for keratoconus to coexist with endothelial dysfunction [32]. A PK is required in these instances.

In 2004, keratoconus was the most common indication for corneal transplantation, but the cases of keratoconus have obviously decreased (Fig 3). This began with the use of collagen crosslinking (CXL) as an effective therapeutic option for progressive keratoconus [33]. Two studies evaluated the effects of CXL on decreasing the number for keratoplasty procedures in patients with keratoconus. First, Godefrooij et al. reported a significant decrease in the need for keratoplasty after the implementation of CXL [34]. The other study showed that the need for keratoplasty in the treatment of keratoconus has been cut in half, mostly by the introduction of the CXL treatment [35].

The scarcity of corneal grafts is a common dilemma over most of the world. In the last few years, several authors have showed different approaches to decrease the shortage of corneal tissue. For example, Lam et al. reported the “hemi-DMEK” for increasing the pool of endothelial graft tissue [36]. In addition, Heindl et al. suggested the split cornea transplantation for two recipients [5], and Yoeruek et al. reported the double-split keratoplasty [4]. One donor cornea may be used for the transplantation in a double-split keratoplasty: a DALK in 1 patient and 2 halved DMEKs with larger diameters in 2 additional patients as a split-DMEK, respectively. This technique could help restore vision in three recipients. Thus far, we have not yet routinely applied these methods, but we are considering them in the near future.

Potential future alternatives to EK that could help address the not fulfilled demand for donor corneas and reduce the growing waiting list include removing central guttae and regenerating a central endothelial cell layer from healthy peripheral cells in patients with Fuchs' dystrophy [37] or injecting cultured human corneal endothelial cells into the anterior chamber to rehabilitate corneas without residual healthy endothelium and recover of the corneal transparency [38,39].

## Conclusions

Our research data showed major changes in the preferred corneal transplantation techniques and leading indications for keratoplasty over the last 12 years. Most importantly, it seems that the rapid development and implementation of EK, especially the DMEK, has had a profound effect on and begun a new era in corneal transplantation.
